# Protein Bodies in Leaves Exchange Contents through the Endoplasmic Reticulum

**DOI:** 10.3389/fpls.2016.00693

**Published:** 2016-05-23

**Authors:** Reza Saberianfar, Amirali Sattarzadeh, Jussi J. Joensuu, Susanne E. Kohalmi, Rima Menassa

**Affiliations:** ^1^Agriculture and Agri-Food CanadaLondon, ON, Canada; ^2^Department of Biology, University of Western OntarioLondon, ON, Canada; ^3^Department of Molecular Biology and Genetics, Cornell UniversityIthaca, NY, USA; ^4^VTT Technical Research Centre of FinlandEspoo, Finland

**Keywords:** protein body, protein body formation, protein trafficking, zera, elastin-like polypeptides (ELP), hydrophobin (HFBI), *Nicotiana benthamiana*, confocal microscopy

## Abstract

Protein bodies (PBs) are organelles found in seeds whose main function is the storage of proteins that are used during germination for sustaining growth. PBs can also be induced to form in leaves when foreign proteins are produced at high levels in the endoplasmic reticulum (ER) and when fused to one of three tags: Zera®, elastin-like polypeptides (ELP), or hydrophobin-I (HFBI). In this study, we investigate the differences between ELP, HFBI and Zera PB formation, packing, and communication. Our results confirm the ER origin of all three fusion-tag-induced PBs. We show that secretory pathway proteins can be sequestered into all types of PBs but with different patterns, and that different fusion tags can target a specific protein to different PBs. Zera PBs are mobile and dependent on actomyosin motility similar to ELP and HFBI PBs. We show *in vivo* trafficking of proteins between PBs using GFP photoconversion. We also show that protein trafficking between ELP or HFBI PBs is faster and proteins travel further when compared to Zera PBs. Our results indicate that fusion-tag-induced PBs do not represent terminally stored cytosolic organelles, but that they form in, and remain part of the ER, and dynamically communicate with each other via the ER. We hypothesize that the previously documented PB mobility along the actin cytoskeleton is associated with ER movement rather than independent streaming of detached organelles.

## Introduction

In plants, seeds offer highly specialized organelles for protein storage. These include oil bodies (OBs), protein storage vacuoles (PSVs) and protein bodies (PBs). Several classes of storage proteins are found in seeds; albumins (water soluble), globulins (dilute saline soluble), prolamins (alcohol soluble), and glutelins (dilute acid or base soluble) (Shewry et al., 1995). Prolamins are the major class of proteins found in cereals such as maize, rice and wheat, and are generally deposited in the endoplasmic reticulum (ER) as dense accretions termed protein bodies (PBs) (Pompa and Vitale, 2006). PBs generally form within the ER lumen but they may also bud off and remain in the cytosol or may be taken up by PSVs through autophagy (Levanony et al., 1992).

Because of their ER origin and their significant role in protein storage, PBs represent important targets for storing recombinant proteins. Therefore, several attempts have been made to target recombinant proteins to seed PBs (Arcalis et al., 2004; Takaiwa et al., 2009; Takagi et al., 2010). Leaves don't naturally have PBs, but overexpression of certain proteins caused the appearance of novel organelles reminiscent of PBs which offer an ideal compartment for storing recombinant proteins in an otherwise proteolytic environment. Protein production in leaves allows for harvesting before flowering, thus reducing the possibility of gene leakage to the environment through pollen and seeds (Conley et al., 2011).

PB formation in leaves can be induced by high levels of heterologous proteins and by the use of fusion tags. We have shown that PB formation initiates when a recombinant protein accumulates at or above 0.2% of total soluble protein (TSP) (Gutiérrez et al., 2013; Saberianfar et al., 2015). This process is not limited to a specific protein, but addition of fusion tags facilitated PB formation (Saberianfar et al., 2015). Three types of fusion tags were shown to enhance PB formation in leaves: Zera®, elastin-like polypeptide (ELP) and hydrophobin-I (HFBI). Zera is derived from the N-terminal region of γ-zein, a prolamin usually found in maize, and consists of a signal peptide with no prolines and a “CGC” motif, a proline-rich region containing eight “PPPVHL” repeats, and a sequence with four cysteine residues. Cysteine residues enable disulfide bond formation between Zera molecules, and the amphipathicity of the (PPPVHL)_8_ region helps the self-assembly and therefore facilitates the ordered packing of Zera molecules (Torrent et al., 2009b; Llop-Tous et al., 2010).

ELPs are synthetic polypeptides composed of “VPGXG” pentapeptide repeats found in mammalian elastin proteins (Urry and Parker, 2002). These repeats form β-helices responsible for the aggregation of ELP chains. Solubility and aggregation of ELP strands rely heavily on temperature, salt concentration, chain length, and the degree of ionization of the guest amino acid (Xaa) (Floss et al., 2010). HFBI belongs to a family of amphipathic globular proteins found in filamentous fungi. All hydrophobins contain eight cysteine residues in their sequence which form four intramolecular disulfide bridges responsible for the self-assembly and aggregate formation of HFBI molecules (Hakanpää et al., 2006).

PBs associated with the three tags have different physical characteristics; Zera-induced PBs are more electron dense compared to ELP or HFBI PBs (Torrent et al., 2009b; Conley et al., 2011), and ELP-induced PBs are larger in size compared to HFBI PBs (Saberianfar et al., 2015). The process by which fusion tag-induced PBs are formed in leaves has been studied to some extent. In Zera, both the proline-rich repeat region, and the two N-terminal cysteine residues were found to be essential for PB formation (Llop-Tous et al., 2010). We have previously shown that ELP and HFBI-associated PB formation is a non-selective mechanism dependant on recombinant protein concentration both in stable transgenic lines and in transient expression (Gutiérrez et al., 2013; Saberianfar et al., 2015), and that proteins targeted to the secretory pathway are sequestered in ELP- and HFBI-induced PBs (Saberianfar et al., 2015).

Here, we asked whether PBs originate from the ER, bud off and become terminally stored cytosolic organelles or remain in the ER as communicating subdomains. To investigate PB biogenesis, we co-expressed the tags with each other or with secretory and ER-targeted proteins, and we tracked PB movement and communication. We hypothesize that PBs form on the ER, move with the ER, and communicate with each other through the ER.

## Materials and methods

### Construct design and cloning

Secretory GFP, GFP, GFP-ELP (Conley et al., 2009a), GFP-HFBI (Joensuu et al., 2010), RFP-HFBI, and RFP-ELP (Saberianfar et al., 2015) plant expression vectors were previously published. Zera-DsRed, GFP-SQS, and CFP-SQS were generously provided by Dr. Dolors Ludevid (Joseph et al., 2012). Zera-EGFP was synthesized by Bio Basic Canada (Markham, ON, Canada).

### Transient expression in *N. Benthamiana* leaves

*N. benthamiana* plants were grown at 22°C with a 16 h photoperiod at a light density of 110 μmol m^−2^ s^−1^ for 7 weeks before infiltration. Plants were watered with the water soluble fertilizer (N:P:K = 20:8:20) at 0.25 g/L (Plant Products, Brampton, ON, Canada). *Agrobacterium tumefaciens* cultures were prepared as previously described (Saberianfar et al., 2015). All the treatments were performed in the presence of p19, a suppressor of gene silencing (Silhavy et al., 2002), to ensure high accumulation levels.

### Tissue sampling and protein extraction

*N. benthamiana* leaf samples were collected at 4 days post infiltration (dpi). Four leaf discs were collected from three biological replicates per treatment. Protein extraction and total soluble protein quantification was performed as previously described (Conley et al., 2009a).

### Recombinant protein quantification

Quantification of EPO was performed by sandwich ELISA as previously described (Conley et al., 2009b). Four biological replicates were quantified per treatment. *N. benthamiana* leaf tissue infiltrated with p19 was used as control.

### Confocal microscopy and image analysis

To visualize the leaf samples, the abaxial epidermal cells were imaged with a Leica TCS SP2 CLSM. GFP was imaged by excitation with a 488 nm argon laser and detection at 500–525 nm. RFP and DsRed were imaged by excitation with 543 nm He/Ne laser and detection at 553–630 nm and 550–600, respectively.

DsRed, CFP, and GFP sequential imaging was performed with an Olympus LSM FV1200. DsRed and GFP were imaged as described above. CFP was excited at 440 nm and its emission was collected at 450–485 nm.

Photoconversion experiments were performed with either a Zeiss LSM 510 confocal microscope or an Olympus LSM FV 1200. The settings for the Zeiss LSM 510 were as follows; a 405 nm laser (50 mW at 100% power setting) was used for photoconversion. PB photoconversion was performed as described in Sattarzadeh et al. (2015) by using between 20–40% of the laser power with 30–40 iterations. Green-state GFP was imaged by excitation at 488 nm and detection at 500–525 nm, whereas red-state GFP was excited by 543 nm laser and detected at 580–640 nm. To visualize the movement of proteins *in vivo*, multiple iterations with time intervals were used to image the trafficking of new GFP into the region of irradiation. Images were processed with the Zeiss Zen software version 6.1.7601. Z-stack confocal images were used to generate 3D images and videos by using Imaris® software (version 7.6.1, Bitplane AG, Switzerland). All the photoconversion experiments were repeated with at least three biological replicates. The settings for the Olympus microscope were as follows; a 405 nm laser (50 mW at 10% power setting for 700 ms) was used for photoconversion. Green- and red-state GFP were imaged as described above.

In co-expression experiments all images were acquired at 4 dpi. We used the sequential mode to avoid crosstalk between the fluorescent channels.

### Latrunculin-B treatment

For actin depolymerization experiments, leaves were infiltrated with a 25 μM solution of Latrunculin-B (Sigma-Aldrich, Saint Louis, MO, USA). Drug treatment was performed 1 h before visualization. The working solution of Latrunculin-B was diluted in distilled water from a 10 mM stock prepared in dimethyl sulfoxide (DMSO).

### Statistical analysis

Minitab Express™ software (Minitab Inc., PA, USA) was used to perform the statistical analysis. The Kolmogorov-Smirnov test was used to confirm normal distribution of the data. A one-way analysis of variance (one-way ANOVA) was performed followed by Tukey-Kramer's test to find significant differences (statistical difference was defined as *p* < 0.05).

## Results

### Secretory pathway proteins are sequestered into the core of ELP- or HFBI-induced PBs, but only to the periphery of zera PBs

**Figure 1:**
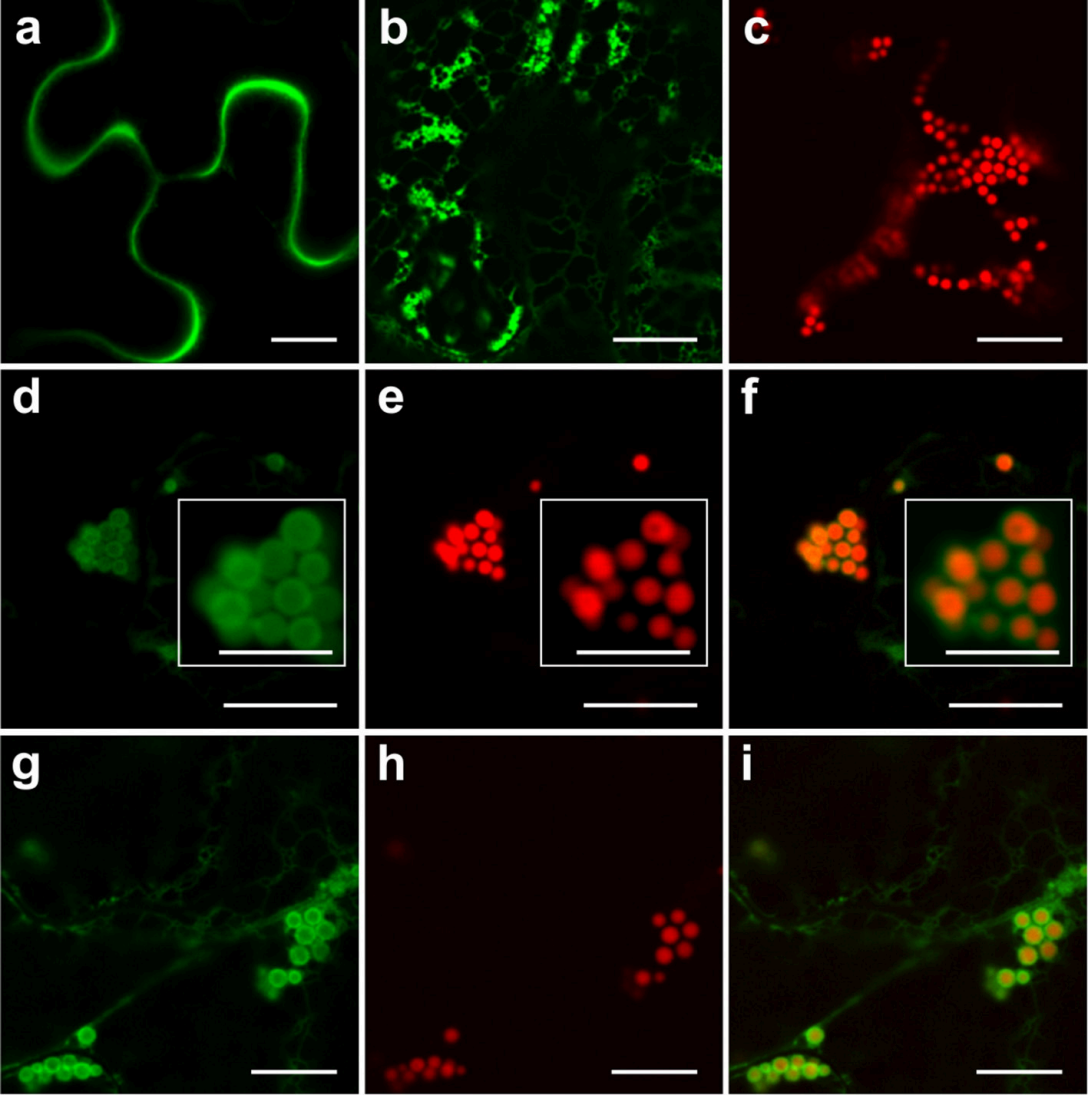
**Secretory and endoplasmic reticulum-targeted GFP are sequestered in Zera-induced PBs. (A)** Secretory GFP highlights the apoplast between cells. **(B)** ER-targeted GFP forms small PBs along the ER network. **(C)** Zera-DsRed induces the formation of PBs. **(D–F)** Co-expression of secretory GFP and Zera-DsRed results in localization of secretory GFP to the periphery of Zera-DsRed PBs. White boxes show close-ups of the PB cluster. **(G–I)** Co-expression of ER-targeted GFP and Zera-DsRed results in localization of GFP to the periphery of Zera-DsRed PBs. All images were acquired at 4-dpi in sequential mode. Bar, 10 and 5 μm in white boxes **(D–F)**.

**Figure 2:**
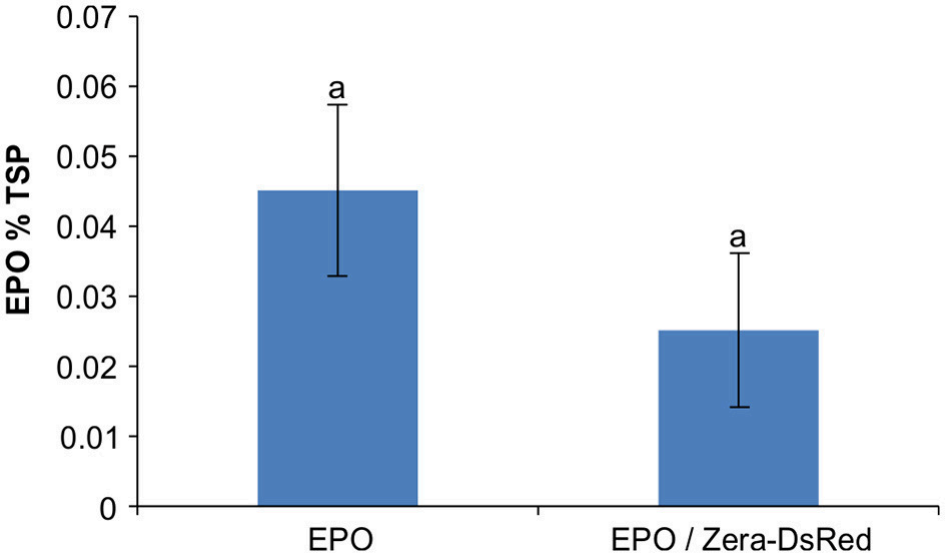
**Co-expression of erythropoietin and Zera-DsRed**. Enzyme-linked immunosorbent assay (ELISA) of recombinant EPO was used for quantification of EPO in agroinfiltrated *N. benthamiana* leaves. Each column represents the mean value of 4 biological replicates collected at 4-dpi. Columns denoted with the same letter were not significantly different (*p* < 0.05) using one-way ANOVA. The error bars represent the standard deviation of the mean.

A previous study of PBs induced by ELP and HFBI showed sequestration of secretory and ER-targeted proteins into the lumen of PBs when co-expressed with ELP or HFBI in *Nicotiana benthamiana* leaves. This property of PBs was used as a tool to increase accumulation levels of difficult-to-express proteins such as erythropoietin (EPO) and interleukin-10 (IL-10) (Saberianfar et al., 2015). A proteomic study of Zera-induced PBs showed the presence of secretory pathway proteins in isolated PBs as well (Joseph et al., 2012). To test if recombinant secretory proteins can also be trapped in Zera-DsRed PBs, we co-expressed secretory GFP or ER-targeted GFP (GFP-KDEL) with Zera-DsRed in *N. benthamiana*. When expressed alone, secretory GFP localizes to the apoplast between the cells (Figure 1A) while ER-targeted GFP highlights the ER and induces the formation of very small PBs along the ER network (Figure 1B), and Zera-DsRed gives rise to distinct PBs (Figure 1C). Upon co-expression of secretory GFP and Zera-DsRed, secretory GFP is found in Zera-induced PBs, but it does not mix with Zera-DsRed which localizes to the center of PBs; instead, GFP is seen at the periphery of PBs (Figures 1D–F). Co-expression of ER-targeted GFP and Zera-DsRed showed a similar pattern with GFP-KDEL mostly surrounding Zera-DsRed (Figures 1G–I; Supplementary Movie 1). Since recombinant fluorescent proteins targeted to the secretory pathway appeared to localize to Zera-induced PBs, we co-expressed EPO with Zera-DsRed to test for an increase in accumulation of EPO in *N. benthamiana* leaves similar to results reported previously for ELP and HFBI PBs (Saberianfar et al., 2015). We found that accumulation of EPO co-expressed with DsRed did not significantly change compared to EPO expressed alone (Figure 2). These results demonstrate a major difference between Zera-induced PBs and ELP or HFBI-induced PBs, both in how other proteins localize in the PBs, and in the effect on accumulation of other proteins, and lead us to further investigate differences between these PBs.

### ELP- and HFBI-fused proteins can be targeted to the same PBs unlike zera-fused proteins

**Figure 3:**
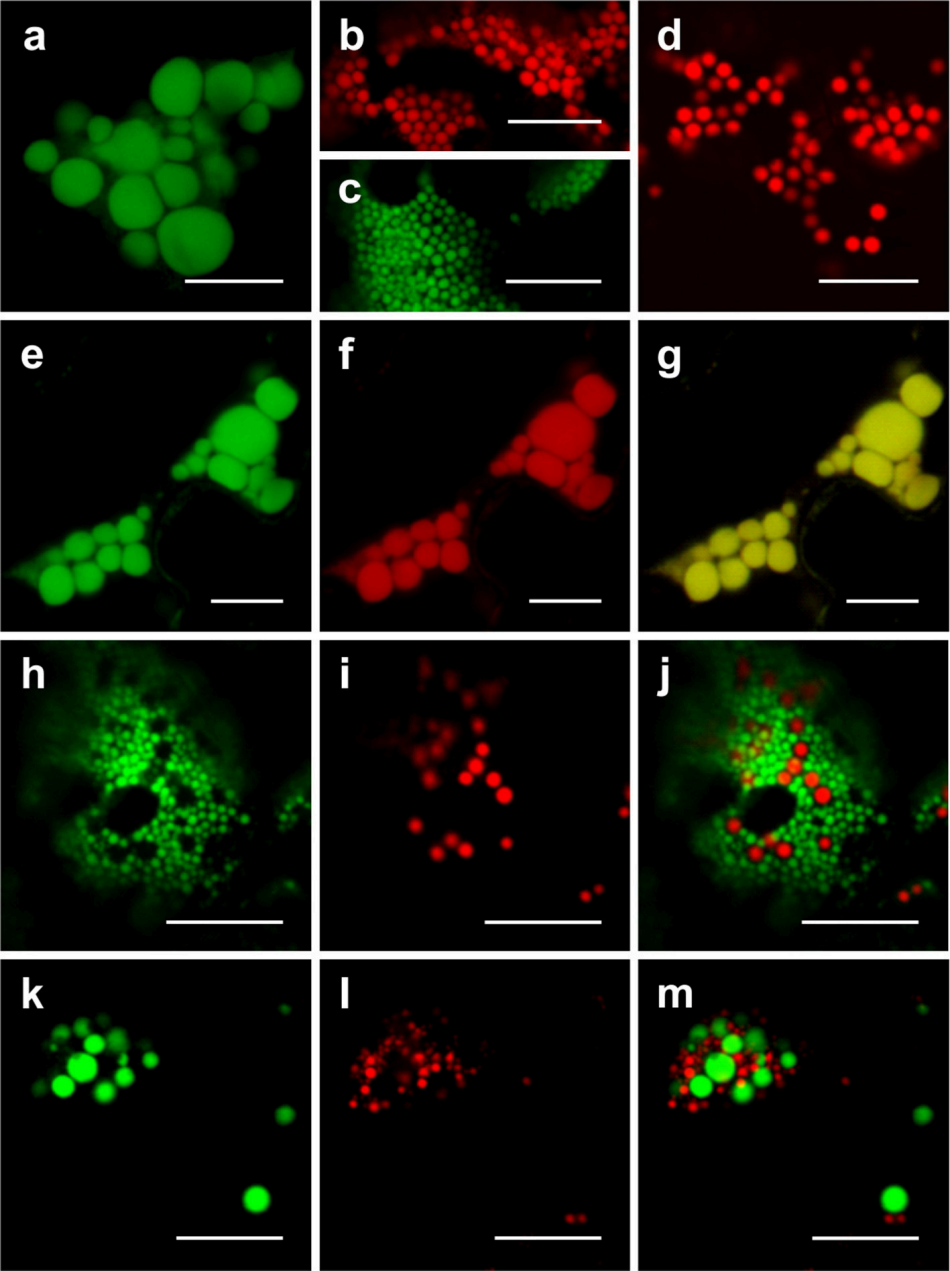
**Co-expression of ELP-, HFBI-, and Zera-fused fluorescent proteins**. When expressed alone, GFP-ELP **(A)** promotes the formation of clusters of large PBs, RFP-HFBI **(B)**, and GFP-HFBI **(C)** form clusters of small PBs, and Zera-DsRed **(D)** induces the formation of small PBs. **(E–G)** Co-expression of GFP-ELP and RFP-HFBI results in co-localization of the recombinant proteins into the same large PBs. **(H–J)** Co-expression of GFP-HFBI and Zera-DsRed results in distinct PBs. (**K–M)** Co-expression of GFP-ELP and Zera-DsRed results in distinct PBs. All images were acquired at 4-dpi in sequential mode. Bar, 10 μm.

To characterize the relationship or distinctness of Zera-, ELP- and HFBI-induced PBs, co-expression analyses were performed. When GFP-ELP, RFP-HFBI, GFP-HFBI, or Zera-DsRed were expressed alone, each induced the formation of PBs (Figures 3A–D). As shown previously (Saberianfar et al., 2015), ELP-induced PBs were larger in size compared to HFBI- or Zera-induced PBs. Upon co-expression of GFP-ELP and RFP-HFBI, both proteins co-localized into the same PBs (Figures 3E–G). However, co-expression of either fusion tag with Zera did not result in co-localization of the proteins into the same PBs, and gave rise to the formation of separate PBs (Figures 3H–M; Supplementary Movies 2, 3).

### ELP-, HFBI-, and zera-induced protein bodies are surrounded by ER membrane

**Figure 4:**
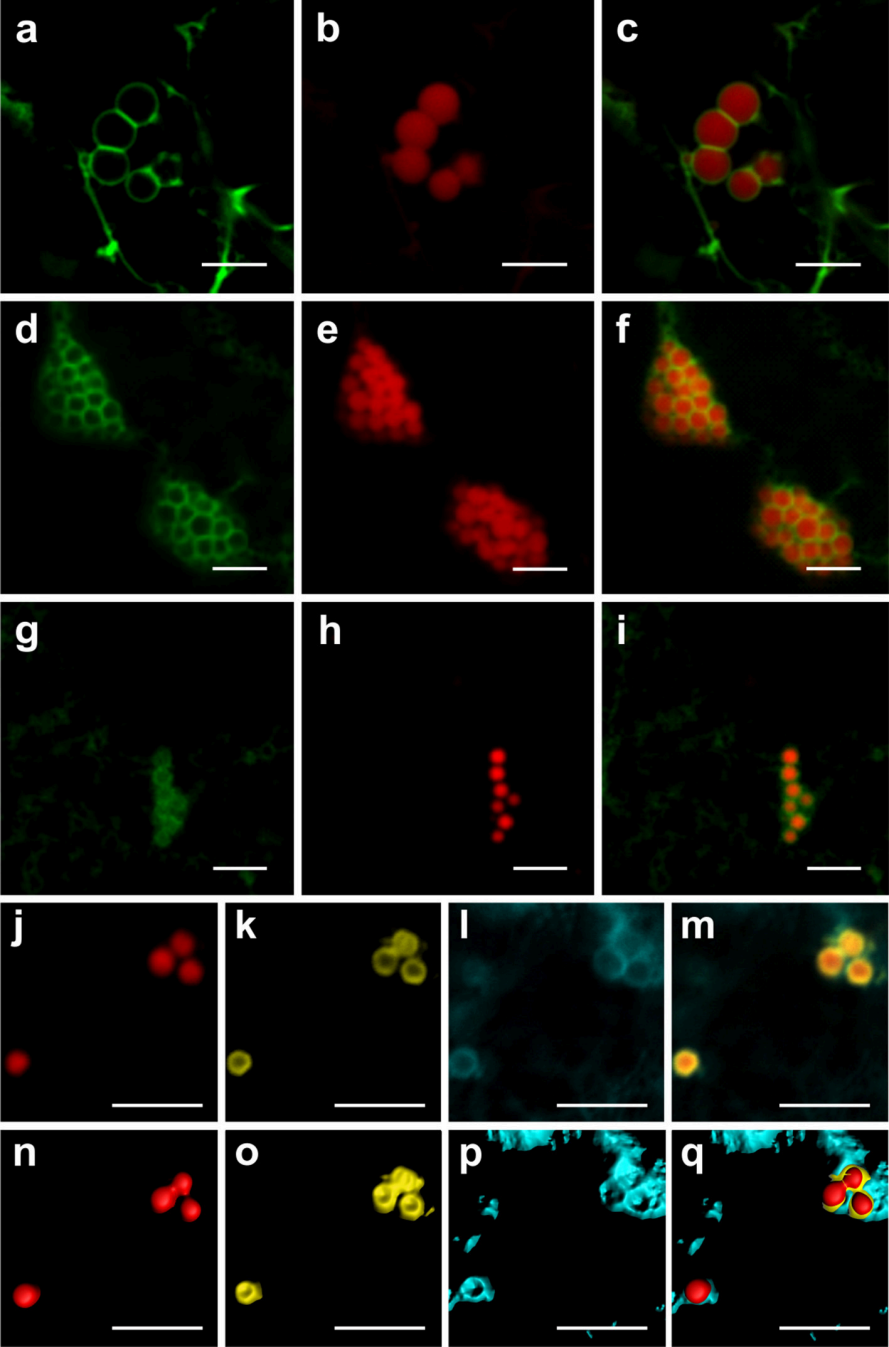
**Protein bodies are surrounded by an endoplasmic reticulum-derived membrane. (A–C)** Co-expression of GFP-SQS1 and RFP-ELP results in the formation of RFP-ELP PBs surrounded with GFP-SQS1 highlighting the ER membrane. **(D–F)** Co-expression of RFP-HFBI and GFP-SQS1 results in PBs surrounded by GFP-SQS1. **(G–I)** Co-expression of Zera-DsRed and GFP-SQS1 results in the formation of Zera-DsRed PBs surrounded by GFP-SQS1. **(J–M)** Zera-DsRed **(J)** localizes to the core of PBs and secretory GFP **(K)** is pushed away from the core to the periphery of PBs. GFP is shown in yellow in this case to allow visualization of the signal. CFP-SQS1 **(I)** highlights the ER membrane. **(M)**. Merge image representing the signals from all three channels shows the ER membrane surrounding both GFP and Zera-DsRed.**(N–Q)** signals from j-m were highlighted in 3D with the Imaris software to allow accurate visualization of each protein. All images were acquired at 4-dpi in sequential mode. Bar, 5 μm.

Previous reports have suggested an ER origin for the fusion-tag-induced PBs in leaves. ELP, HFBI and Zera were shown by transmission electron microscopy to be surrounded by a membrane studded with ribosomes and thought to be an ER-derived membrane (Conley et al., 2009a; Joensuu et al., 2010; Joseph et al., 2012). To ascertain that PBs induced by ELP, HFBI, and Zera originate from the ER, we co-expressed RFP-ELP, RFP-HFBI, and Zera-DsRed with the ER transmembrane domain (C-terminus) of *Arabidopsis thaliana* squalene synthase 1 (SQS1) fused to GFP (GFP-SQS1) at the N-terminus, with GFP facing the cytosolic side of the ER membrane (Kribii et al., 1997). We found that GFP-SQS1 highlights the membrane surrounding PBs induced by RFP-ELP (Figures 4A–C), RFP-HFBI (Figures 4D–F), and Zera-DsRed (Figures 4G–I) upon transient co-expression. In cases where several PBs were aligned side by side, the membrane showed a continuous (uninterrupted) pattern around each PB. It is also important to note that the membrane surrounding PBs appears to be continuous with the ER surrounding the cluster of PBs (Figures 4C,F,I).

Because secretory GFP appeared to surround PBs induced by Zera (Figure 1), we co-expressed CFP-SQS1, secretory GFP and Zera-DsRed. The resulting PBs contained Zera-DsRed in their core, with secretory GFP around the edges within the ER membrane as highlighted by CFP-SQS1 (Figures 4J–Q; Supplementary Movie 4).

### Protein bodies communicate with one another

Previous work had shown that ELP-PBs are mobile and that their movement is dependent on actin microfilaments and myosin motor proteins (Conley et al., 2009a). PBs were also shown to be mobile (Joensuu et al., 2010), but there was no information on the mobility of Zera PBs. We therefore investigated if Zera-EGFP PBs are mobile, and found that they indeed display movement (Supplementary Movie 5). Time lapse imaging of Zera-EGFP PBs in the presence of Latrunculin-B, an inhibitor of actin polymerization, eliminated the PB movement (Supplementary Movie 6) and indicated that Zera PBs are dependent on actin microfilaments for their movement, as shown previously for ELP PBs (Conley et al., 2009a; Joensuu et al., 2010).

ELP and HFBI PBs were hypothesized to bud off the ER and to exist as terminally-stored cytosolic organelles because of their ability to move inside the cell (Conley et al., 2011). However, Conley et al. (2009a) also showed the recovery of fluorescence of GFP-ELP-induced PBs within 5 min after photobleaching suggesting the trafficking of GFP from other parts of the cell to the bleached PB. Similarly, co-expression of YFP-KDEL with Zera-induced PBs revealed localization of YFP to the periphery of Zera-induced PBs (Llop-Tous et al., 2010). Fluorescence recovery after photobleaching (FRAP) resulted in rapid recovery of YFP in these PBs as well as in the ER within 80 s leading the authors to conclude that Zera PBs are connected with the ER. Our results indicating the presence of an ER membrane around PBs prompted us to investigate if PBs bud off completely and leave the ER or if they remain connected with the ER, allowing them to exchange their content with other PBs and the surrounding ER.

A limitation of the FRAP technique is the inability to visualize the bleached protein, leaving unanswered the question of protein trafficking out of or into PBs. An alternative method for observing protein trafficking *in vivo* is photoconversion. We recently discovered the ability of GFP to photoconvert irreversibly from its well-known green-state to red-state upon irradiation with the 405 nm laser (Sattarzadeh et al., 2015). Therefore, we used GFP fusions of HFBI, ELP and Zera to track the protein's movement after photoconversion in a specific PB or a group of PBs.

**Figure 5:**
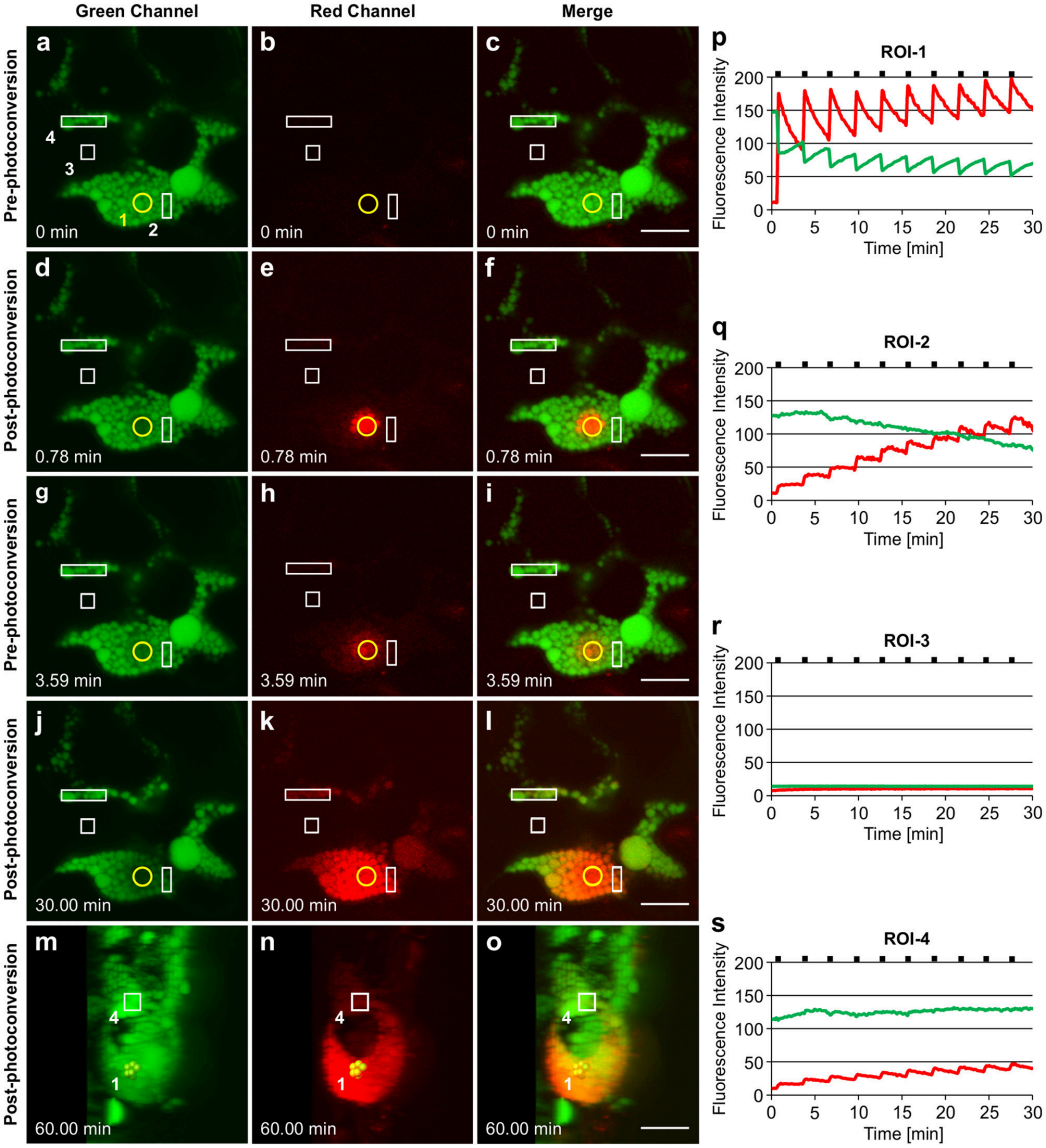
**Trafficking of proteins between GFP-HFBI-induced protein bodies. (A–C)** GFP-HFBI-induced PBs form clusters and can only be visualized in the green channel before photoconversion. The yellow circle represents the region of irradiation (ROI-1). Regions of interest (ROI 2-4) are shown in white rectangles. **(D–F)** Upon irradiation of ROI-1, GFP in PBs within the circle photoconverts to red and starts spreading into neighboring PBs. **(G–I)** Within ~3 min of the first irradiation, GFP recovers at ROI-1 and the red signal is spread to neighboring and distant PBs more than 10 μm away from ROI-1. **(J–L)** After multiple irradiations (using 3 min time intervals) of ROI-1, sufficient red fluorescence is produced that shows the trafficking of the photoconverted protein from ROI-1 to ROI-4 and PBs further away (more than 20 μm from ROI-1). **(M–O)** 3D representation of the PB cluster at a 90° rotation. PBs irradiated at ROI-1 are highlighted in yellow. ROI-4 is shown with a square. PBs between ROI-1 and ROI-4 are linked with a chain of PBs at a different focal plane within the cell. **(P–S)** Changes in fluorescence intensity of the green and red signal in ROI-1 **(P)**, ROI-2**(Q)**, ROI-3 **(R)**, and ROI-4 **(S)**. Black notches indicate the time of irradiation. All images were acquired at 4-dpi in sequential mode. Bar, 10 μm.

Irradiation of GFP-HFBI PBs resulted in immediate photoconversion of the irradiated PBs and their neighboring PBs in and around the region of irradiation-1 (ROI-1) (Figures 5A–F). This is also demonstrated by the sudden decrease of the green fluorescence intensity and the simultaneous increase of the red fluorescence intensity at ROI-1 (Figure 5P). Photoconverted protein (red-state) then spread into the surrounding PBs. Approximately 3 min after the first irradiation, fluorescence intensity of the red signal at ROI-1 was decreased by half (Figure 5P) as the photoconverted red signal was trafficked to the adjacent PBs and whereas GFP increased, presumably due to trafficking to ROI-1 (Figures 5G–I,P). We noticed that by repetitive irradiation of GFP trafficked to ROI-1 with 3 min intervals more photoconverted protein (red signal) could be produced, which enabled us to track the signal as it traveled to neighboring (ROI-2) and distant PBs (ROI-4) located more than 20 μm away from ROI-1 (Figures 5J–L,S) (Supplementary Movie 7). These 3 min intervals were long enough to avoid heating up the tissue and the time-lapse experiment was performed over a 45 min period to avoid any potential artifact caused by repeated irradiations. It is important to note that GFP fluorescence at ROI-1 recovered gradually after every irradiation (Figure 5P). This recovery can be due to trafficking of GFP from neighboring PBs in ROI-2 (Figure 5Q) in which GFP fluorescence gradually decreased over time and was replaced with the red fluorescent signal coming from ROI-1.

Photoconverted protein only traveled to other PBs, and the appearance of the red fluorescent signal in PBs at ROI-4, which did not seem to be connected to ROI-1, can be explained by the presence of a chain of PBs located in a different focal plane (Figures 5M–O; Supplementary Movie 8). ROI-3 was chosen as an area free of the ER since the ER should otherwise be highlighted as the expressed protein contains a KDEL retrieval signal. Considering the absence of changes in either green or red fluorescence intensities at ROI-3, located in the area between ROI-1 and ROI-4 (Figures 5A–L,R), we conclude that trafficking of proteins between PBs only happens either directly through PBs or through ER connections between PBs.

**Figure 6:**
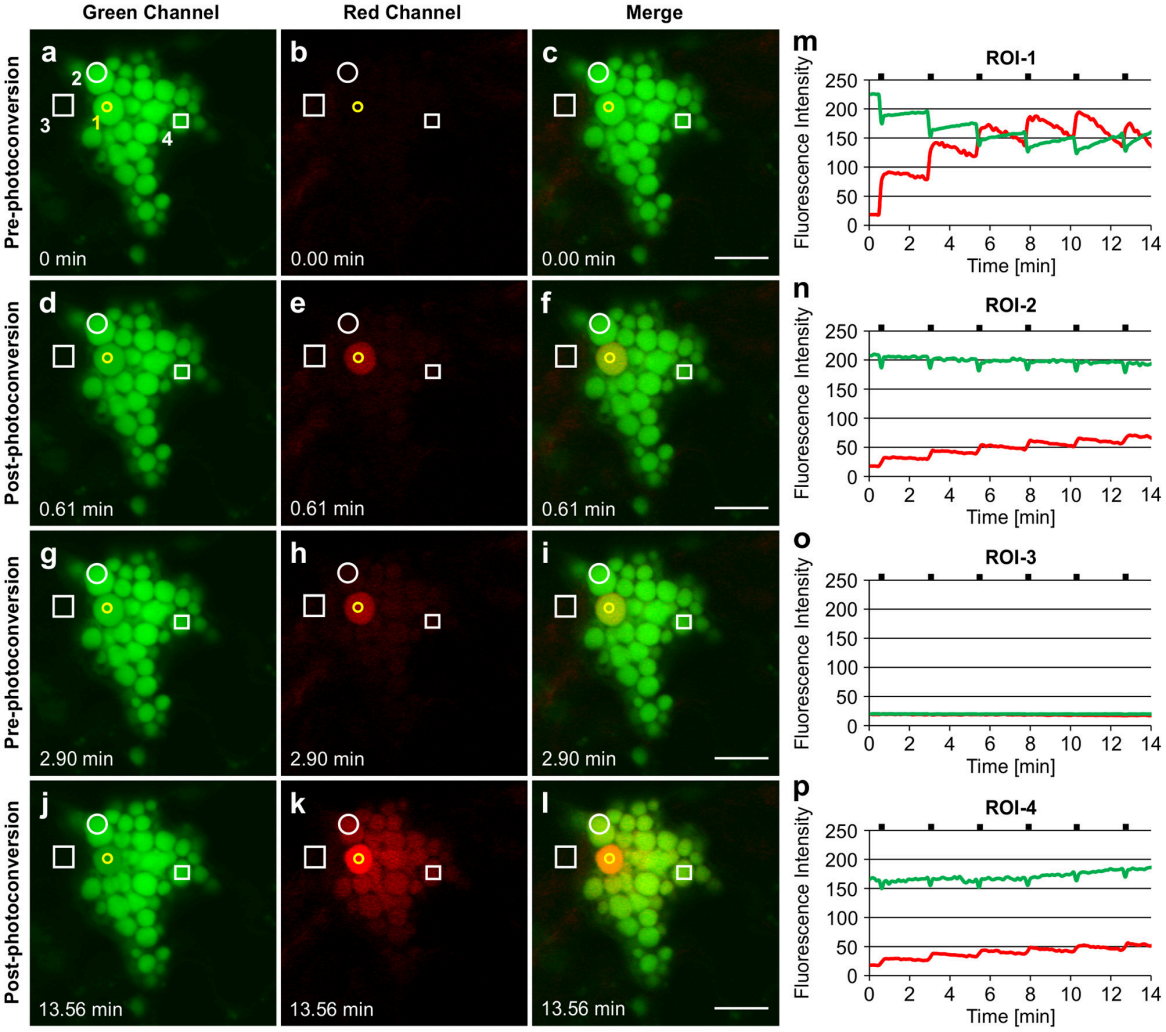
**Trafficking of proteins between ELP-induced protein bodies. (A–C)**. GFP-ELP induced PBs form clusters and can only be visualized in the green channel before photoconversion. The yellow circle represents the region of irradiation (ROI-1). Regions of interest (ROI 2-4) are shown in white. **(D–F)**. Photoconversion of the whole PB upon irradiation of a specific region within a PB. **(G–I)**. Spread of the photoconverted red signal from ROI-1 into the neighboring (ROI-2) and distant (ROI-4) PBs within 2.30 min. (**J–L)**. After multiple irradiations (with 2.30 min time intervals) of ROI-1, sufficient red fluorescence is produced that shows the trafficking of the photoconverted protein from ROI-1 to other PBs. **(M–P)**. Changes in fluorescence intensity of the green and red signal in ROI-1 **(M)**, ROI-2**(N)**, ROI-3 **(O)**, and ROI-4 **(P)**. Notches indicate the time of irradiation. All images were acquired at 4-dpi in sequential mode. Bar, 10 μm.

Similar results were obtained with ELP-induced PBs (Figure 6). Upon irradiation of ROI-1 within a single PB, the entire PB rapidly photoconverted to red fluorescence (Figure 6E; Supplementary Movie 9) suggesting a high mobility of GFP-ELP within PBs. The photoconverted signal gradually spread to neighboring PBs (ROI-2) while the green fluorescence at ROI-1 recovered within approximately 2 min (Figures 6G–I,M,N). We repeated the photoconversion of the fluorescent proteins within ROI-1 with 2.30 min intervals and monitored the trafficking of the red fluorescent signal into neighboring (ROI-2) and distant (ROI-4) PBs (Figures 6J–L,N,P). We also noticed a sudden minor drop in the green fluorescence intensities at ROI-2 and ROI-4 upon irradiation of ROI-1 (Figures 6N,P, respectively). This might be due to immediate trafficking of GFP to the photobleached area at ROI-1 from the other PBs. Monitoring the fluorescence intensity at ROI-3 showed no changes of the green or red fluorescent signal and therefore confirms the trafficking of the photoconverted protein between PBs and not through the cytoplasm (Figures 6A–L,O).

**Figure 7:**
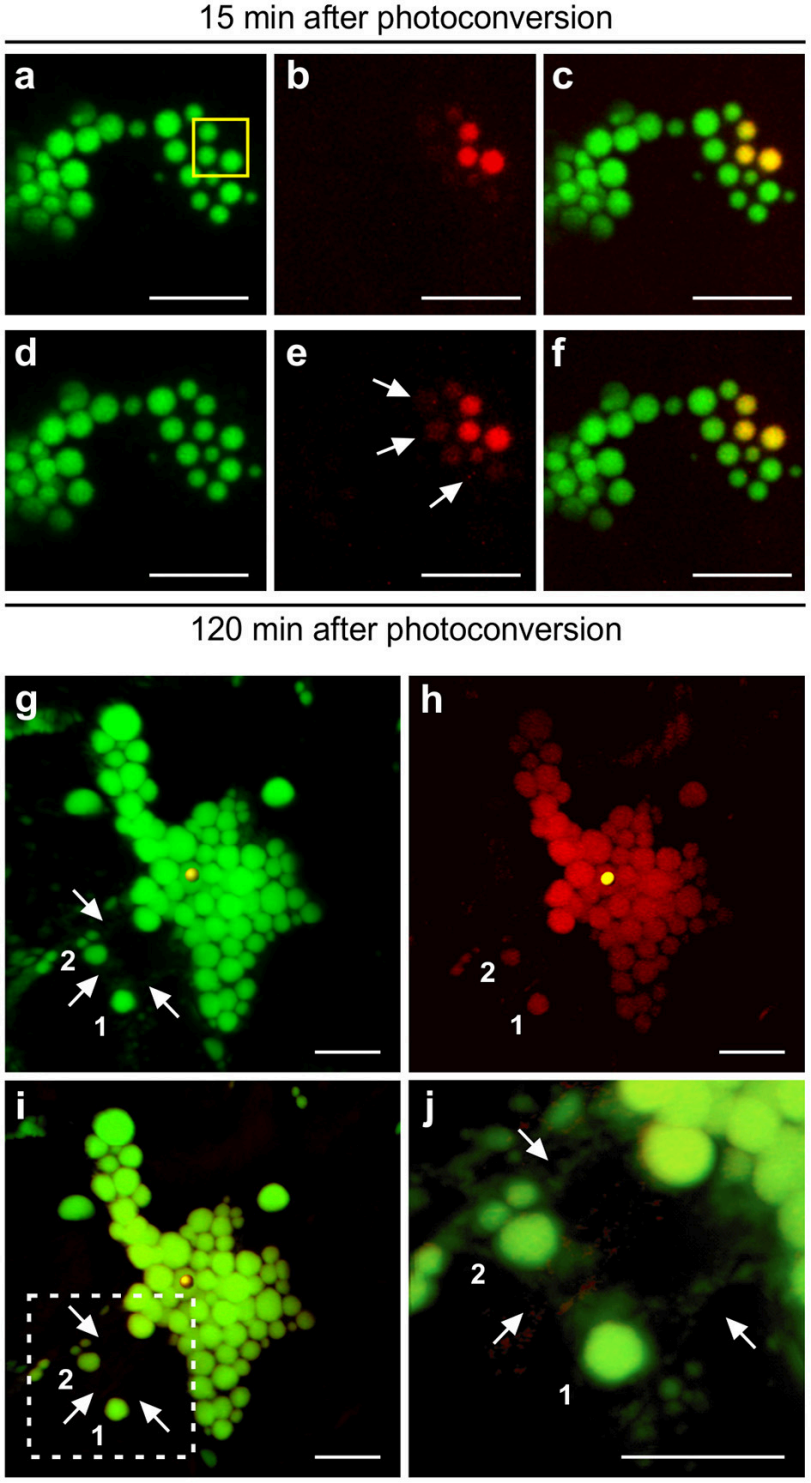
**Protein trafficking in Zera and ELP PBs upon photoconversion. (A–F)** 3D visualization of a cluster of Zera-GFP PBs after photoconversion. **(A–C)** PBs highlighted with yellow box were irradiated twice with 7 min intervals. Z-stack images were acquired 15 min after photoconversion.**(D–F)** Z-stack images were acquired 120 min after the initial photoconversion. A small amount of photoconverted protein is trafficked from the region of irradiation into neighboring PBs shown with arrows.**(G–J)** ELP PBs communicate with one another through the ER. **(G)** 3D visualization of GFP-ELP PBs in the green channel. Region of irradiation is shown by a yellow sphere. PBs marked as 1 and 2 are located away from the region of irradiation and the cluster of PBs, and connected to the rest of PBs only through the ER network shown with arrows. **(H)** 3D visualization of photoconverted GFP-ELP PBs in the red channel. PBs marked as 1 and 2 photoconverted to red even though disconnect from the PB cluster. **(I)** Merge image of the green and red channels. **(J)** Close-up of the dotted square area in **(I)**. Arrows point to the ER network. All images were acquired at 4-dpi in sequential mode. Bar, 10 μm.

In the case of Zera-induced PBs, irradiation of a number of PBs in a cluster (Figure 7A), causes instant photoconversion of green fluorescence to red fluorescence (Figures 7B,C), but spread of the red fluorescence to neighboring PBs was barely visible after 120 min, unlike the quick spread of fluorescence observed for ELP- and HFBI-induced PBs. PBs shown in the yellow box were irradiated twice with 7 min intervals and monitored over a 2 h period after the initial irradiation during which the red fluorescent signal traveled very slowly from the region of irradiation into the neighboring PBs (Figures 7D–F).

**Figure 8:**
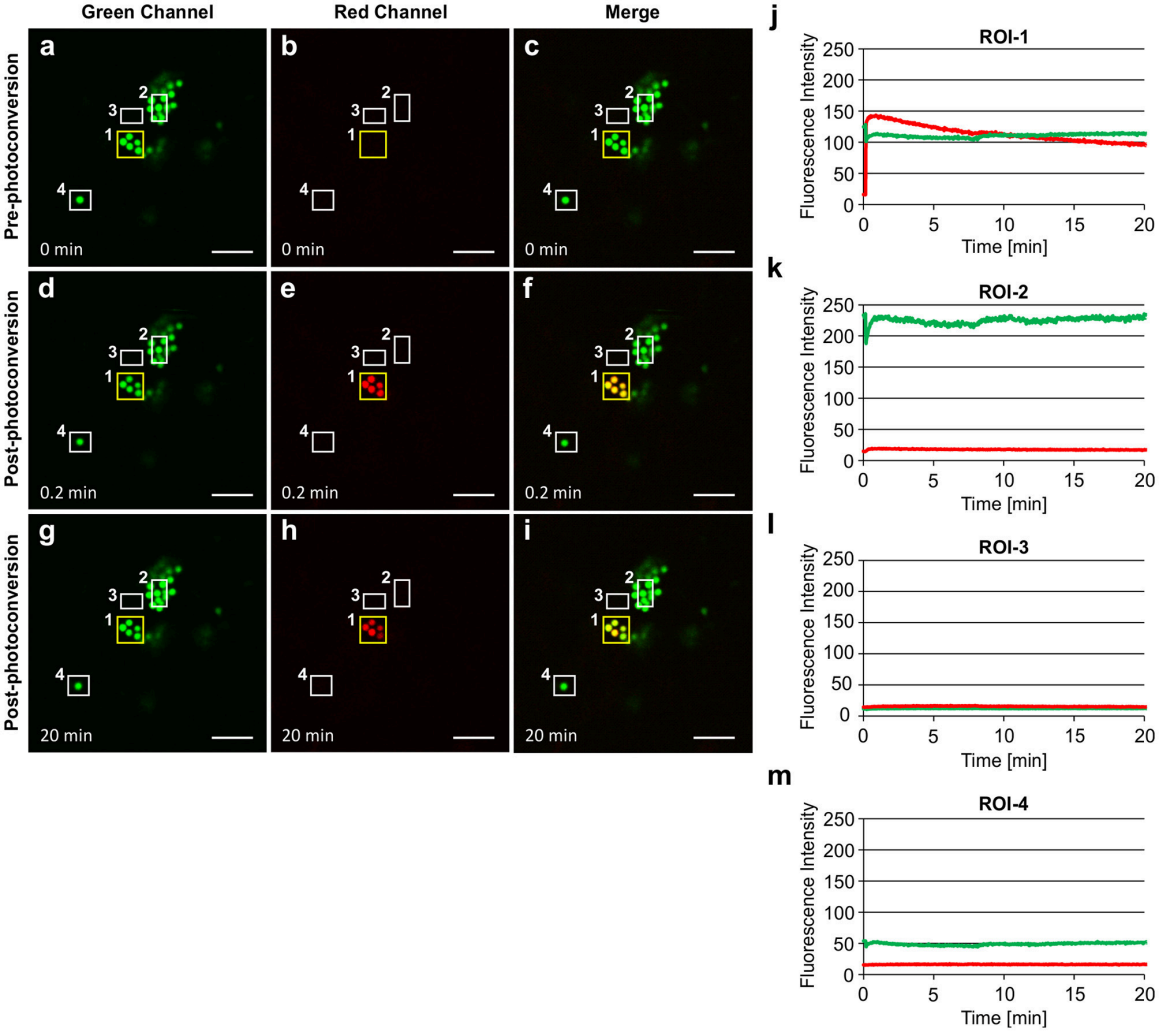
**Zera PB contents are not as soluble as ELP or HFBI PBs**. The yellow box highlights the region of irradiation (ROI-1). Other regions of interests are shown in white boxes (ROI-2-4).**(A–C)** Zera PBs can be seen only in the green channel before photoconversion. **(D–F)** PBs in ROI-1 were irradiated once and turned red within seconds.**(G–I)** The majority of photoconverted red signal remains in the irradiated PBs.**(J–M)** Changes in the fluorescence intensity of the green and red signal in ROI-1 **(J)**, ROI-2 **(K)**, ROI-3 **(L)**, and ROI-4 **(M)**. Imaging was done in the presence of Latrunculin-B to keep PBs in the same focal plane. Notches indicate the irradiation period. All images were acquired at 4-dpi in sequential mode. Bar, 10 μm.

The photoconversion of EGFP-Zera PBs was repeated on multiple independent biological replicates and very little trafficking of the photoconverted protein was observed each time by time-lapse monitoring (Figure 8; Supplementary Movie 10). Upon irradiation of ROI-1, PBs turn to red fluorescence instantly (Figures 8A–F,J), the green fluorescent signal is slightly reduced and remains constant afterwards. The red fluorescent signal remains mostly in the irradiated PBs after 20 min (Figure 8J). The photoconverted red fluorescent signal was very strong which eliminated the need for multiple rounds of irradiation to constantly produce red fluorescence in these PBs, as was necessary in the case of ELP and HFBI PBs. During this time, no red fluorescence appeared in neighboring PBs (ROI-2, and 4) or in the cytoplasm (ROI-3) (Figures 8G–I,K–M). This result is consistent with the properties of the Zera peptide which assembles with intramolecular disulfide bonds in the core of the PBs, and would therefore be less soluble, and less conducive to trafficking than ELP- or HFBI-fused proteins.

### PB communication occurs through the ER network

To understand if protein trafficking occurs via direct connections between PBs or if it occurs through the ER network we analyzed 3D images generated by compilation of Z-stacks of GFP-ELP images acquired 45 min post-photoconversion (Figures 7G–J). As expected, the red fluorescence was observed in all the PBs clustered together at, above and below the focal plane at which the photoconverted PB was located (Figure 7H). We also noticed that a number of PBs located away from the cluster of PBs also became red fluorescent (denoted as 1 and 2 in Figures 7G–J). A high magnification 3D image of these isolated PBs showed that they are surrounded by the ER network which extends around the cluster of PBs (Figure 7J; Supplementary Movie 11). We believe that the ER network provides a bridge through which proteins are trafficked between the PB cluster and isolated PBs.

## Discussion

### Fusion-tag-induced leaf PBs resemble seed PBs but are different enough to have their own category

Several types of protein storage organelles can be found in seeds. In dicotyledonous seeds, proteins are stored in protein storage vacuoles (PSVs). In cereals, proteins are stored in PBs and in PSVs which differ with respect to the storage proteins they contain. PBs originate from the ER and are mostly composed of prolamins, while PSVs form *de novo* and contain albumins and globulins (Shewry and Halford, 2002). The pathways that storage proteins take from the ER to PBs or PSVs are different and complex depending on the plant species (Khan et al., 2012). For instance, albumins and globulins in many plant species traffic from the ER through the Golgi to PSVs via dense vesicles (Hohl et al., 1996) but in pumpkin seeds they bypass the Golgi by forming precursor-accumulating (PAC) vesicles and deliver their contents directly to PSVs (Hara-Nishimura et al., 1998). PB formation in most seeds initiates in the ER lumen and depending on the plant species they might remain within the lumen of the rough ER as dense aggregates or bud off the ER, bypass the Golgi apparatus and be absorbed by PSVs (Galili, 2004; Khan et al., 2012).

In leaves, there are reports of spindle-shaped organelles observed when GFP was targeted to the ER with a C-terminal HDEL retrieval motif, known as fusiform bodies (Gunning, 1998). Fusiform bodies are located in the ER of epidermal and cortical cells (Hawes et al., 2001) and contain high levels of β-glucosidase (Matsushima et al., 2003). The PBs we investigated in this study were all induced by fusion tags (Zera, ELP, or HFBI) and shared a series of similarities: they are high in numbers, round shaped, they form clusters, are surrounded by ER membrane and are not absorbed by the central vacuole, but rather are associated with the ER (at least until 6 dpi; Llop-Tous et al., 2010; Saberianfar et al., 2015). Therefore, we believe that these fusion-tag-induced PBs, although they share some similarities with seed PBs, fusiform bodies or PAC vesicles, should be described as “fusion-tag-induced leaf-based PBs.”

### Proteins targeted to the secretory pathway are sequestered passively into zera-induced PBs

A previous proteomic analysis of Zera PBs has shown the presence of ER resident proteins, such as BiP, calnexin and calreticulin, and secretory proteins such as cell wall proteins in Zera-induced PBs (Joseph et al., 2012). In agreement with these results, we found that upon co-expression of secretory GFP or GFP-KDEL with Zera, GFP was sequestered into Zera-induced PBs. Interestingly, GFP was restricted to the periphery of PBs, which is different from the even distribution of secretory GFP or GFP-KDEL in ELP or HFBI PBs (Saberianfar et al., 2015). Nevertheless, even though being localized to the periphery of PBs, it seems as if proteins targeted to the secretory pathway are sequestered passively in Zera-induced PBs, as is the case for ELP and HFBI-induced PBs (Saberianfar et al., 2015). It is possible that the physico-chemical properties of Zera prevent other recombinant proteins from penetrating into the core of PBs. This observation is in agreement with the study of molecular dynamics of Zera by Llop-Tous et al. (2010) in which they proposed that Zera molecules exhibit a sticklike conformation, and that the amphipathicity of the (PPPVHL)_8_ repeat regions of Zera imposes lateral protein-protein interaction among Zera molecules and therefore hydrophobic packing of Zera-PBs. This might be the reason why GFP is pushed away from the core of Zera PBs and seen as circles surrounding Zera-DsRed even though GFP was still localized to the PB lumen surrounded with an ER membrane.

### Co-expression of low accumulating proteins with zera-induced PBs is not as efficient as with ELP- or HFBI-induced PB

It is thought that PBs protect recombinant proteins from degradation and also protect the cell from potential toxic effects of high levels of recombinant proteins (Conley et al., 2011). Indeed, co-expression of proteins targeted to the secretory pathway with PB-inducing fusion tags such as ELP and HFBI was found to increase accumulation levels of low accumulating proteins such as EPO and IL10 (Saberianfar et al., 2015). To test if this is a universal phenomenon, in this study we co-expressed EPO with a PB-inducing Zera fusion. Unlike the previous results with ELP and HFBI fusions, we did not observe an increase in EPO accumulation levels. We hypothesize that the physico-chemical properties of Zera prevent efficient integration of EPO molecules into the lumen of Zera-induced PBs, similar to what we observed with co-expression of GFP and Zera-DsRed. Therefore, even though secretory proteins can incorporate into Zera PBs, the capacity of integration into Zera PBs is limited compared to ELP or HFBI PBs due to the strong affinity between Zera molecules.

### Proteins can be targeted to different PBs

We found that ELP and HFBI fusion proteins co-localize to the same PBs, while neither of them co-localizes with Zera-induced PBs. The conventional KDEL signal peptide is bound by the ERD2 receptor on the cis-Golgi that retrieves proteins back to the ER (Lewis et al., 1990; Napier et al., 1992). Considering that both ELP and HFBI fusion proteins were retrieved to the ER in this fashion and that Zera-fused proteins accumulate in the ER and form PBs without the need for an ER retrieval signal suggests the possibility that Zera PBs may originate from a separate ER subdomain (Staehelin, 1997; Choi et al., 2000; Hamada et al., 2003; Lynes and Simmen, 2011). It is also possible that Zera molecules behave differently compared to ELP and HFBI due to their very strong affinity toward one another. Zera is a derivative of γ-zein which belongs to a family of maize seed storage proteins called prolamins. In seeds, prolamins are known to induce PBs by forming large aggregates in the ER due to their hydrophobicity and disulfide bond formation. It was suggested that these prolamin-induced aggregates are excluded from transport to the Golgi complex by COP-II vesicles, due to their large size and therefore induce PB formation (Vitale and Ceriotti, 2004; Kawagoe et al., 2005; Pompa and Vitale, 2006). The hydrophobic region of Zera molecules was shown to enable lateral protein-protein interactions which result in stick-like alignment of Zera molecules. This structure is additionally stabilized by intermolecular disulfide bonds formed between cysteine residues (Llop-Tous et al., 2010). These features contribute to PB formation and may prevent integration of other proteins into the core of Zera PBs. For instance, Llop-Tous et al. (2010) were able to introduce GFP into the core of Zera-induced PBs only when they fused GFP to a Zera fusion tag. Therefore, the affinity of Zera molecules to each other might prevent the integration of ELP- or HFBI-fused proteins to Zera PBs. In addition, the nature of Zera may be incompatible with the hydrophobic nature of ELP and HFBI proteins (Linder, 2009; Floss et al., 2010), and therefore, Zera localizes to separate PBs. This feature of fusion proteins can potentially be used for simultaneous expression of different proteins *in vivo* and their targeting to the same or separate PBs. The PB-associated proteins can then be specifically purified by applying tag-specific purification techniques. While ELP and HFBI proteins can be purified using non-chromatographic strategies specific to their physico-chemical properties, Zera PBs can be purified by density gradient centrifugation (Linder et al., 2001; Urry and Parker, 2002; Torrent et al., 2009b).

### Protein bodies remain part of the ER and communicate with one another

Zera PBs are believed to originate from the ER, grow in size and remain within the ER (Torrent et al., 2009a; Llop-Tous et al., 2010). On the other hand, ELP and HFBI PBs were suggested to bud off the ER and become terminally stored in the cytosol. This assumption was made based on the presence of membranes studded with ribosomes surrounding ELP and HFBI PBs, that PBs did not appear to be connected with the ER in electron micrographs, and because they were seen to be mobile, to move along the actin cytoskeleton, and their movement to be dependent on intact actin microfilaments (Conley et al., 2009a; Joensuu et al., 2010). The dependence of PBs movement on the actin cytoskeleton was shown by disruption of their rapid movement when co-expressed with a dominant negative mutant of myosin XI-K tail, and by treatment with Latrunculin B which depolymerizes actin (Conley et al., 2009a).

Here, we show that all PBs form and align along ER strands, are surrounded by an ER membrane, and that the movement of PBs induced by Zera is disrupted by the use of Latrunculin B, similar to previous observations with Latrunculin B treatment of ELP PBs (Conley et al., 2009a). We also show that PBs located far away in different focal planes exchange content through the ER if tracked long enough after photoconversion. Therefore we believe that PBs are protein aggregations that form and remain within the ER, and that their movement is associated with ER movement.

The ER is a highly dynamic organelle especially in plants. Rapid ER movement is caused by classical cytoplasmic streaming, whereby ER movement is controlled by dynamic interactions between three components; ER, actin, and myosin (Yokota et al., 2011; Griffing et al., 2014; Hawes et al., 2015; Ueda et al., 2015). Interestingly, Peremyslov et al. (2012) showed that in Arabidopsis leaves, the majority of the ER is myosin-free but only a motile subdomain of the ER, mostly composed of ER-derived vesicles, is associated with myosin. This is in agreement with our observations of PB movement. It is in fact possible that our PBs are similar to the “ER-derived vesicles” observed by Peremyslov et al. (2012) and that those vesicles are similarly associated with the ER.

Protein trafficking between PBs was previously suggested by Conley et al. (2009a) when studying GFP-ELP-induced PBs upon photobleaching in FRAP experiments, although the reason for the recovery was not clear and was attributed to either trafficking from other PBs or synthesis by ribosomes found on the PB membrane. We used the recently developed technique of green to red photoconversion of GFP (Sattarzadeh et al., 2015) to show the trafficking of proteins between PBs. Our results with ELP and HFBI PBs confirmed that these PBs exchange their content with each other rapidly after photoconversion (within seconds) via the ER. Conversely, trafficking of the photoconverted proteins out of the irradiated PBs happened much slower with Zera PBs (within hours). We believe the low solubility of Zera may be the reason why Zera fusions display slower/less protein trafficking compared to ELP and HFBI PBs.

In summary, we found that fluorescence recovery of all photoconverted PBs occurred through protein trafficking from neighboring PBs, but we cannot fully rule out the possible role of *de novo* protein synthesis by ribosomes available on the PB membranes in recovery of PB contents (Conley et al., 2009a; Joensuu et al., 2010; Llop-Tous et al., 2010).

### Working model of active exchange of proteins between PBs and PB movement

**Figure 9:**
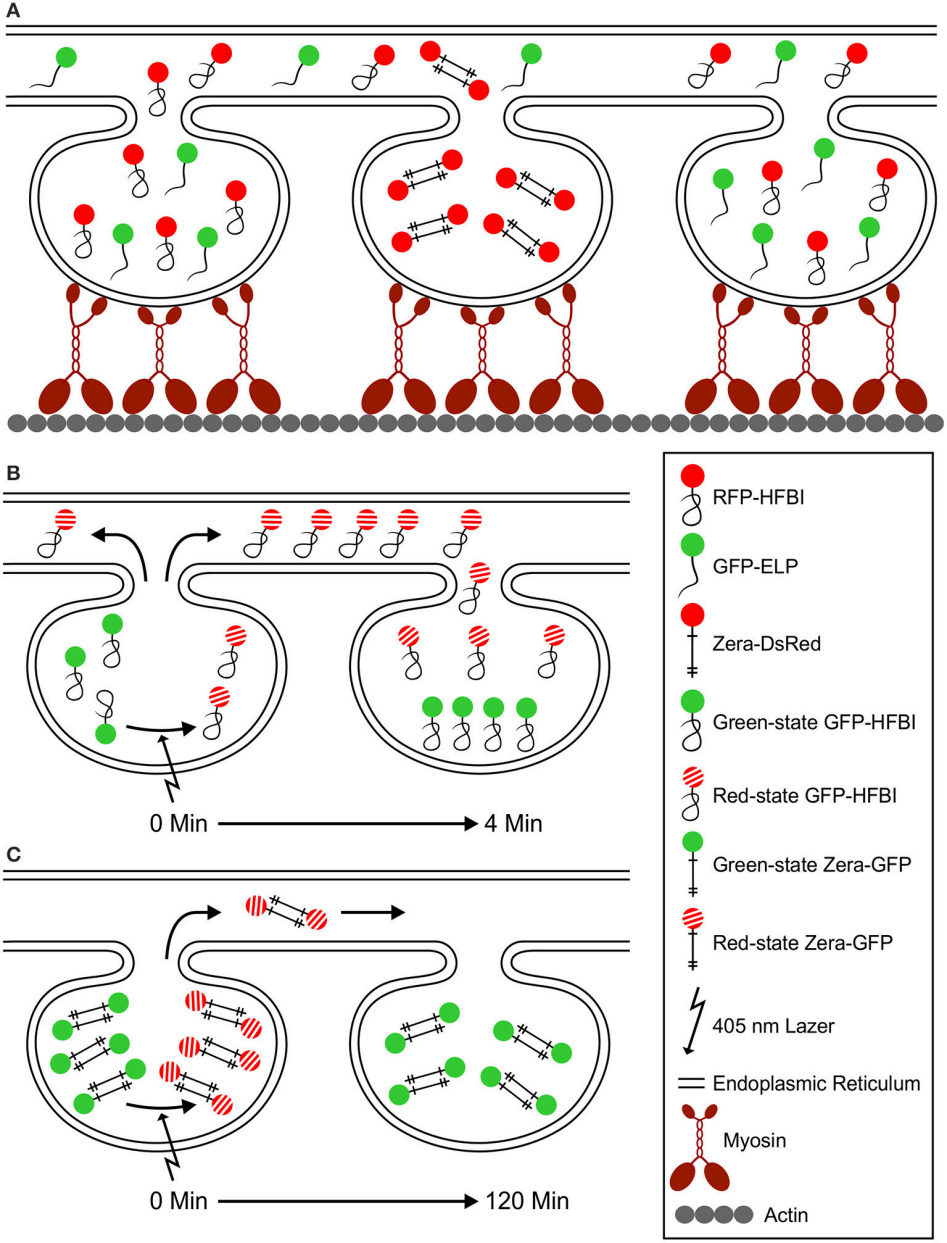
**A model for PB movement and active exchange of PB content via the ER. (A)** Zera, ELP and HFBI PBs are surrounded with an ER membrane and are mobile *in vivo*. Myosin motor proteins attach to the ER with their globular tail domain and to actin filaments with their motor domain (Peremyslov et al., 2012). PB mobility depends on ER movement at contact points with the actomyosin cytoskeleton. Upon co-expression of different protein fusions, ELP and HFBI fused proteins co-localize to the same PBs, but not with Zera-fused proteins. This is due to the high affinity of Zera molecules to one another that is the result of intermolecular disulfide bridges between cysteine residues (shown with crosses). **(B)** Upon photoconversion of GFP from green- to red-state, GFP-HFBI molecules travel (in < 4 min) from one PB to another through the ER. ELP fusion proteins act similar to HFBI fusions upon photoconversion (not shown in this model). **(C)** Zera-GFP molecules do not fold after synthesis, preserve their sticklike structure, and form intermolecular disulfide bonds, and therefore are less mobile (soluble) compared to HFBI or ELP protein fusions. By photoconverting the content of Zera-GFP PBs, they immediately change from green to red state, but the movement from one PB to another happens in a very slow fashion when compared to HFBI and ELP protein fusions. Very little protein movement is observed 120 min after photoconversion.

Based on our observations, we hypothesize that PBs form bulges in the ER lumen, remain connected to the ER and do not form detached organelles in the cytosol. PBs rely on the ER and the actomyosin cytoskeleton for their movement. Myosin molecules attach to the ER membrane around PBs on one side with their globular tail domains and to actin strands on the other side with their motor domain. Myosin proteins traveling on actin drag the ER and PBs within the ER (Figure 9A). ELP and HFBI fusion proteins co-localize to the same PBs unlike Zera fusions. This is because Zera fusion proteins do not fold and instead preserve their sticklike conformation, form intermolecular disulfide bonds, and have high affinity to each other, which does not allow the integration of other proteins into their core (Figure 9A). Proteins traffic between PBs via the ER (Figures 9B,C), and ELP and HFBI fusions traffic rather rapidly from one PB to a neighboring PB (Figure 9B) compared to Zera fusions (Figure 9C).

## Author contributions

RS designed the research, performed the experiments and wrote the manuscript. RM conceived the study and participated in its design and wrote the manuscript. AS assisted with the photoconversion experiments. JJ provided feedback on experimental design and result interpretations. RS, AS, SK, and RM edited the manuscript.

### Conflict of interest statement

The authors declare that the research was conducted in the absence of any commercial or financial relationships that could be construed as a potential conflict of interest.

## Supplementary material

Supplementary Movie 1 **Endoplasmic reticulum-targeted GFP is localized at the periphery of Zera-induced PBs**. Z-stack images of cells co-expressing ER-targeted GFP and Zera-DsRed were acquired by confocal microscopy and assembled into a 4D illustration using the Imaris software. ER-targeted GFP is localized to the periphery of Zera-induced PBs and also highlights the ER. Zera PBs were visible in the red channel. Simultaneous visualization of both green and red channels shows that ER-targeted GFP surrounds Zera-DsRed PBs. The signals in green and red channels were detected with the Imaris software and highlighted to show the accurate localization of each signal. Z-stack images were acquired at 4 dpi in sequential mode. Bar, 5 μm.

Supplementary Movie 2 **Co-expression of HFBI- and Zera-fused fluorescent proteins**. Co-expression of GFP-HFBI and Zera-DsRed induced the formation of separate PBs. GFP-HFBI PBs were visualized in the green channel and Zera-DsRed were detected in the red channel. Simultaneous visualization of both channels showed HFBI and Zera PBs as separate PBs. Z-stack images were acquired by confocal microscopy and used for 4D illustrations. Green and red signals were detected and highlighted with the Imaris software to show the accurate localization of PBs. Z-stack images were acquired at 4 dpi in sequential mode. Bar, 5 μm.

Supplementary Movie 3 **Co-expression of ELP- and Zera-fused fluorescent proteins**. Co-expression of GFP-ELP and Zera-DsRed induced the formation of separate PBs. GFP-ELP PBs were visualized in the green channel and Zera-DsRed were detected in the red channel. Simultaneous visualization of both channels showed ELP and Zera PBs as separate PBs. Z-stack images were acquired by confocal microscopy and used for 4D illustrations. Green and red signals were detected and highlighted with the Imaris software to show the accurate localization of PBs. Z-stack images were acquired at 4 dpi in sequential mode. Bar changes from 3-10 μm depending on the magnification.

Supplementary Movie 4 **Co-expression of Zera-DsRed, and secretory GFP with an ER membrane marker**. Co-expression of Zera-DsRed with secretory GFP and CFP-SQS1shows the localization of Zera-DsRed to PB core that is immediately surrounded by secretory GFP. The ER membrane highlighted by CFP-SQS1 wraps around secretory GFP. Z-stack images were acquired by confocal microscopy and used for 4D illustrations. Red, green and blue were detected and highlighted with the Imaris software to show the detailed localization of each signal. Imaging was performed at 4 dpi in sequential mode. Bar, 1 μm.

Supplementary Movie 5 **Zera-GFP protein bodies move *in vivo***. Time-lapse confocal imaging shows the movement of Zera-GFP PBs in a cell during 25 min. Images were acquired in a *N. benthamiana* leaf treated with 25 μM DMSO solution as a negative control at 4 dpi. Bar, 10 μm.

Supplementary Movie 6 **Trafficking of Zera-GFP protein bodies depend on actin microfilaments**. A *N. benthamiana* leaf was treated with 25 μM Latrunculin-B solution, an actin depolymerizing drug, for 1 h. Time-lapse confocal imaging during 12 min shows that the movement of PBs has stopped due to lack of intact actin microfilaments. Images were acquired at 4 dpi. Bar, 10 μm.

Supplementary Movie 7 **Trafficking of proteins between GFP-HFBI-induced protein bodies**. Time-lapse confocal imaging represents the photoconversion and trafficking of GFP-HFBI between PBs. The yellow circle represents the region of irradiation (ROI-1) which was irradiated with 405 nm laser at 20% of laser power and 40 iterations every 3 min. The white rectangular and square boxes represent the control regions described in Figure 4. Photoconverted GFP-HFBI (red state) can be seen in the red channel. Simultaneous photoconversion of GFP-HFBI from green to red can be easily seen in the merge channel. Images were acquired at 4 dpi. Bar, 10 μm.

Supplementary Movie 8 **GFP-HFBI traffics to distant protein bodies via the endoplasmic reticulum**. GFP-HFBI-induced PBs and the ER are shown approximately 1 h post-photoconversion. The irradiated PBs were detected with the Imaris software and highlighted with yellow spheres. The green channel represents GFP-HFBI. The red channel shows photoconverted GFP-HFBI (red-state). The white box represents the area in which ROI-3 (from Figure 5) was positioned. White arrows highlight the ER in different locations in the cell. Images were acquired at 4 dpi. Bar, 8 μm.

Supplementary Movie 9 **Trafficking of proteins between GFP-ELP-induced protein bodies**. Time-lapse confocal imaging represents the photoconversion and trafficking of GFP-ELP between PBs. The yellow circle represents the region of irradiation (ROI-1) which was irradiated with 405 nm laser at 40% of laser power and 30 iterations every 2.30 min. The white rectangular and square boxes represent the control regions described in Figure 6. Photoconverted GFP-ELP (red state) can be seen in the red channel. The green channel represents GFP-ELP. Simultaneous photoconversion of GFP-ELP from green to red can be easily seen in the merge channel. Images were acquired at 4 dpi. Bar, 10 μm.

Supplementary Movie 10 **Minimal trafficking of Zera-GFP proteins out of PBs after photoconversion**. Time-lapse confocal imaging shows the photoconversion of Zera-GFP PBs. Box number 1 highlights the region of irradiation (ROI-1) that was irradiated with 405 nm laser at 10% of laser power for 700 ms. Boxes number 2–4 represent the control regions (ROI-2 to 4) described in Figure 7. Images were acquired at 4 dpi. Bar, 10 μm.

Supplementary Movie 11 **GFP-ELP traffics to distant protein bodies via the endoplasmic reticulum**. GFP-ELP-induced PBs are shown approximately 45 min post-photoconversion. The irradiated region within the PB was detected with the Imaris software and highlighted with a yellow sphere. The green channel represents the GFP-ELP. The red channel shows photoconverted GFP-ELP (red-state). Note the faint ER network surrounding the PB cluster, and that turns red upon photoconversion. Images were acquired at 4 dpi. Bar, 10 μm.

## Abbreviations

CFP: cyan fluorescent protein
dpi: days post infiltration
EGFP: enhanced green fluorescent protein
ELISA: enzyme-linked immunosorbent assay
ELP: elastin-like polypeptide
EPO: erythropoietin
ER: endoplasmic reticulum
HFBI: hydrophobin-I
IL-10: interleukin-10
KDEL: ER retrieval signal
OB: oil body
OD: optical density
PB: protein body
PSV: protein storage vacuole
RFP: red fluorescent protein
SQS1: squalene synthase 1
TSP: total soluble protein
YFP: yellow fluorescent protein.
